# Antibiotic prescriptions and risk factors for antimicrobial resistance in patients hospitalized with urinary tract infection: a matched case-control study using the French health insurance database (SNDS)

**DOI:** 10.1186/s12879-021-06287-1

**Published:** 2021-06-14

**Authors:** Marion Opatowski, Christian Brun-Buisson, Mehdi Touat, Jérôme Salomon, Didier Guillemot, Philippe Tuppin, Laurence Watier

**Affiliations:** 1 Epidemiology and Modeling of bacterial Evasion to Antibacterials Unit (EMEA), Institut Pasteur, 25–28, Rue du Dr. Roux, 75724 Paris Cedex 15, France; 2 Center for Research in Epidemiology and Population Health (CESP), INSERM U1018, Paris-Saclay University, UVSQ, Montigny-Le-Bretonneux, France; 3 AP-HP, Paris Saclay, Public Health, Medical Information, Clinical Research, Le Kremlin-Bicêtre, France; 4 French National Health Insurance (Cnam), 50 Avenue du Pr-André-Lemierre, 75986 Paris Cedex 20, France

**Keywords:** Antimicrobial resistance, Urinary tract infections, SNDS, Medico-administrative database, Administrative claims database, Risk factors, antibiotic consumption

## Abstract

**Background:**

Antibiotic resistance is increasing among urinary pathogens, resulting in worse clinical and economic outcomes. We analysed factors associated with antibiotic-resistant bacteria (ARB) in patients hospitalized for urinary tract infection, using the comprehensive French national claims database.

**Methods:**

Hospitalized urinary tract infections were identified from 2015 to 2017. Cases (due to ARB) were matched to controls (without ARB) according to year, age, sex, infection, and bacterium. Healthcare-associated (HCAI) and community-acquired (CAI) infections were analysed separately; logistic regressions were stratified by sex.

**Results:**

From 9460 cases identified, 6468 CAIs and 2855 HCAIs were matched with controls. Over a 12-months window, the risk increased when exposure occurred within the last 3 months. The following risk factors were identified: antibiotic exposure, with an OR reaching 3.6 [2.8–4.5] for men with CAI, mostly associated with broad-spectrum antibiotics; surgical procedure on urinary tract (OR 2.0 [1.5–2.6] for women with HCAI and 1.3 [1.1–1.6] for men with CAI); stay in intensive care unit > 7 days (OR 1.7 [1.2–2.6] for men with HCAI). Studied co-morbidities had no impact on ARB.

**Conclusions:**

This study points out the critical window of 3 months for antibiotic exposure, confirms the impact of broad-spectrum antibiotic consumption on ARB, and supports the importance of prevention during urological procedures, and long intensive care unit stays.

**Supplementary Information:**

The online version contains supplementary material available at 10.1186/s12879-021-06287-1.

## Background

Urinary tract infections (UTIs) are one of the most common infections. In the United States, UTIs are estimated to account for 8.6 million healthcare visits annually with associated costs of 1.6 billion dollars in 2007 [1].

Antibiotic resistance rates are increasing among urinary pathogens, both in community and hospital infections, leading to increased therapeutic difficulties, worse clinical and economic outcomes, and longer hospitalizations [2–4]. For example, the proportion of extended-spectrum beta-lactamases-producing (ESBL-p) Enterobacteriaceae among UTI isolates has increased dramatically within the past two decades, with rates up to 10% among *Escherichia coli* isolates, reaching 15% or more among hospital *Klebsiella pneumonia* isolates [5–7].

A number of risks factors for UTI caused by antibiotic-resistant bacteria (ARB) have been described in previous studies, such as age, previous hospitalization, presence of urinary catheter, nursing home residency, previous UTI, exposure to antibiotics, or some co-morbidities such as diabetes or immunosuppression [8–18]. However, these studies mostly focused on ESBL-p Enterobacteriaceae in community or hospital-acquired infections, and based their estimation on a restricted number of cases, with monocentric designs [9–11].

The importance of “real-world data”, derived from registries, electronic health records or administrative databases, that complement data from conventional clinical trials in regulatory decision making process, has been recently emphasized [19]. In France, the national health data system (SNDS) provides access to all hospitalizations, drug dispensing and medical care [20]. Since 2014, antibiotic resistance is identifiable among infections in hospitalized patients, thus allowing to study resistant bacteria in hospital [21, 22]. This study aims to assess the risk factors for infection due to ARB in patients hospitalized for a urinary tract infection, using data from the SNDS.

## Methods

### Data source and collection

The SNDS is a comprehensive anonymized database composed of the national hospital discharge database (*PMSI, Programme de médicalisation des systèmes d’information*), chained with individual patient information and all French outpatient healthcare refund using a pseudomysed identification number, which enables to follow a patient and to identify some co-morbidities [20]. Supplement S1 provides a detailed description of available information (Additional file 1).

Incident hospital admissions for UTI in adults (≥18-years-old), occurring between Jan 1st, 2015 and Dec 31, 2017, and due to an *Escherichia coli*, *Klebsiella pneumoniae*, *Staphylococcus aureus, Pseudomonas aeruginosa* or Enterococci were identified from the PMSI database, using codes lists established in collaboration with infectious diseases specialists (Additional file 1, Table S1). Only stays for which a bacterial species could be associated with the UTI were included [22], and incident hospitalizations then selected, by excluding stays with a previous hospitalization with a UTI code within the past 12 months.

Infections were classified in two categories: healthcare-associated (HCAI), with at least one hospitalization ≥24 h in the preceding 3 months (all causes, without UTI); and community-acquired (CAI), with no hospitalization during this period [23]. Infections due to ARB were identified using specific codes (Additional file 1, Table S1 and S2). Those due to non-resistant bacterium, called in this study susceptible bacterium, were identified when none of the antibiotic resistance codes covered by ICD-10 was filled.

### Risk factors

The following potential risks factors were extracted from the linked databases: i) outpatient antibiotic exposure; ii) urinary tract conditions; iii) associated health conditions; and iv) for HCAI, previous hospitalization in an intensive care unit (ICU).

Identification of several factors required using an algorithm combining different codes (diagnoses from hospitalizations, chronic disease, drug consumption, laboratory tests or surgical procedures) (See Additional file 1, Supplement S2). In the database, diagnoses and chronic diseases are coded using the 10th revision of the International Statistical Classification of Diseases and Related Health Problems (ICD-10) [21]. Drugs are identified through two classification systems: anatomical therapeutic chemical (ATC) and French presentation identification code (CIP, *Code Identifiant de Présentation*) systems, which provides further details about the drug, such as conditioning. Laboratory tests are registered with the French nomenclature of procedures (NABM, *nomenclature des actes de biologie médicale*). Procedures performed both for inpatients or outpatients are coded with the French classification of clinical procedures (CCAM, *Classification commune des actes médicaux*).

#### Antibiotic exposure

Antibiotic exposure was defined using antibiotic refund, according to the ATC code list (Additional file 1, Table S3). In order to exclude antibiotics prescribed for the index UTI, dispensing during the week before hospitalization were not considered. The time interval between antibiotic dispensing and index stay (at least one dispensing in the 8 days─3 months; > 3 months) and the number of dispensing during the last 12 months (0; 1; 2; ≥3) were considered (Additional file 1, Table S4). Because number of antibiotic dispensing and time interval are strongly correlated, a variable combining the two information was created. In addition, the last dispensing in the last 3 months and its association with an outpatient UTI was given particular attention. Four classes of exposures were considered: narrow or broad spectrum antibiotic and associated or not with an UTI (antibiotics spectrum detailed in Table 1). If several antibiotics were delivered simultaneously, the broadest spectrum was considered. Lastly, an exploratory analysis was conducted on a restricted sample regarding the impact of various ATC3 antibiotic classes delivered in the last 3 months. Penicillins were divided in two categories, broad and narrow spectrum, and cephalosporins were isolated from “Other betalactams”. For interpretation clarity, patients with several classes of antibiotic delivered during this period (except the three most frequentcouples) and antibiotics rarely dispensed (< 1% of studied sample) were excluded from this exploratory analysis. When a patient was excluded, so was his/her matched case or control.

**Table 1:** Antibiotic spectrum

	Anatomical Therapeutic Chemical codes	
	Narrow spectrum	Broad spectrum
Tetracycline		J01AA
Amphenicols		J01BA
Beta-lactams and penicillin	J01CE; J01CF	J01CA; J01CG; J01CR
Other beta-lactams	J01DF	J01DB; J01DC; J01DD; J01DE J01DH; J01DI
Sulfonamides and trimethoprim		J01EA; J01EB; J01EC; J01EE
Macrolides, Lincosamides, Streptogramines	J01FA; J01FF; J01FG	
Aminoglycoside		J01GA; J01GB
Quinolone	J01MB	J01MA
Association of antibacterial		J01RA
Other antibacterial	J01XA; J01XB;; J01XX	J01XC; J01XD; J01XE; J01XX01

#### Urinary tract conditions

Antecedents of outpatient UTI in the previous 12 months were identified using an algorithm (Additional file 1, Supplement S2). UTIs recorded during the week before hospitalization were not considered. Recurrent UTIs were defined as ≥3 UTIs recorded within the last 12 months. Prior hospitalization for an urinary tract or renal disease in the previous 12 months, such as kidney failure, bladder tumor, or urinary stone were defined from their ICD-10 codes (Additional file 1, Table S3). Urinary tract surgical procedures were identified using a CCAM code list (Additional file 1, Table S3). The most recent procedure was recorded, and classified according to two characteristics: anatomical location (nearby or in contact with urinary tract), and time interval to the index hospitalization (≤ 3, 3–12 months).

#### Associated health conditions

Diabetes was defined using the French National Health Insurance (Cnam) identification algorithm. Immunosuppression was defined using an algorithm established in collaboration with clinicians. Neurologic pathologies and pregnancy were defined using Cnam algorithms (Additional file 1, Supplement S2).

#### Previous hospitalization in intensive care unit (ICU)

ICU stays > 7 days occurring within the 3 months preceding the index hospitalization were identified for HCAI.

### Analyses

A matched 1:1 case–control approach was used to identify risk factors. Hospitalizations of cases, having infection due to an ARB, were matched with hospitalizations of controls (infection due to an antibiotic-susceptible bacterium), according to patient’s age (±5 years), sex, infection code, year of admission, and bacterial species. CAI and HCAI were studied separately, and analyses were stratified by sex.

First, cases and controls were described, for each category of infection (CAI, HCAI) and by sex. Then, univariate and multivariate conditional logistic regressions were conducted. For multivariate analysis, only factors associated with the risk of acquiring UTI with ARB were included in the model. Two models were built, one considering overall antibiotic exposure, and one focusing on the last antibiotic dispensing.

The same methodology was applied for studying the impact of the class of antibiotics delivered in the previous 3 months.

Because of the large size of the sample, crude (ORc) and adjusted (ORa) odds ratios are reported with their 95% confidence intervals (95% CI) without providing *p*-values [24].

Since risk factors might differ according to bacteria or infection site, interactions between these characteristics and risk factors were tested in the different models.

Statistical analyses were computed with SAS Enterprise Guide (v 7.13 software, SAS Institute Inc., Cary, NC, USA). Data extraction and analysis were approved by the French Data Protection Agency (CNIL, approval DE-2016–176). All methods were performed in accordance with CNIL regulations and with REporting of studies Conducted using Observational Routinely-collected Data (RECORD) guideline.

## Results

### Descriptive analyses

**Fig. 1:**
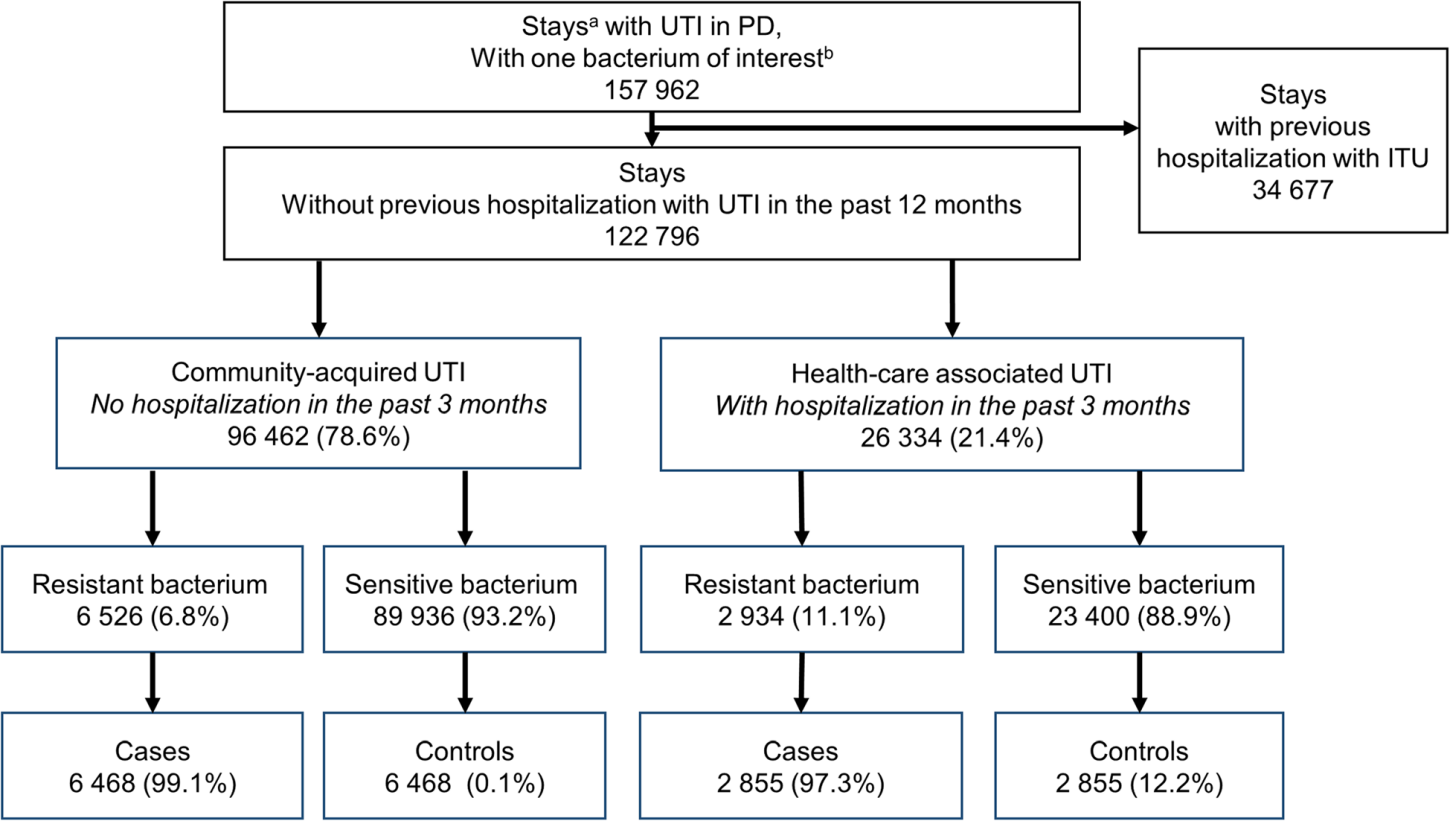
Flowchart of patient selection. Abbreviations: *PD* Principal diagnosis, *UTI* Urinary tract infection

Among 157,962 stays with an UTI and a bacterium of interest identified, 78.0% were incident hospitalizations (Fig. 1). Over the 3-year period, 9460 infections with ARB were identified, including 6526 (69.0%) CAI and 2934 (31.0%) HCAI; 98.5% were matched to infection with a sensitive bacterium, yielding 6468 and 2855 pairs, respectively.

In CAIs, 40.7% of patients were men, in contrast with HCAIs, where men represented more than half of patients (Table 2). In CAIs, the three most frequent infection diagnoses in men were prostatitis, unspecified UTI and tubulo-interstitial nephritis, and tubulo-interstitial nephritis, unspecified UTI, and cystitis in women, representing altogether more than 90% of the CAIs. For both sex, infections were mainly caused by *Escherichia coli* and *K. pneumoniae* (75.8 and 13.3% for men and 87.2 and 9.1% for women, respectively).

**Table 2:** General characteristics of the cases and matched controls by category of infection and gender

	Community-acquired infection				Healthcare-associated infection^a^			
	Men (*N* = 5268)		Women (*N* = 7668)		Men (*N* = 3170)		Women (*N* = 2540)	
	Cases	Controls	Cases	Controls	Cases	Controls	Cases	Controls
Age (years)								
Mean (SD)	71.1 (15.2)	71.1 (15.2)	67.8 (22.0)	67.8 (22.0)	75.1 (12.6)	75.1 (12.6)	74.5 (17.3)	74.5 (17.3)
Median (IQ)	73.0 (21.0)	73.0 (21.0)	75.0 (33.0)	75.0 (33.0)	77.0 (18.0)	77.0 (18.0)	80.0 (21.0)	80.0 (21.0)
Diagnoses								
Tubulo-interstitial nephritis	368 (14.0)	368 (14.0)	2351 (61.3)	2351 (61.3)	266 (16.7)	266 (16.7)	677 (53.3)	677 (53.3)
Pyelonephritis	23 (0.9)	23 (0.9)	92 (2.4)	92 (2.4)	10 (0.6)	10 (0.6)	33 (2.6)	33 (2.6)
Renal and perirenal abscess	55 (2.1)	55 (2.1)	136 (3.5)	136 (3.5)	39 (2.5)	39 (2.5)	39 (3.1)	39 (3.1)
Cystitis	95 (3.6)	95 (3.6)	359 (9.4)	359 (9.4)	91 (5.7)	91 (5.7)	151 (11.9)	151 (11.9)
Unspecified UTI	527 (20.2)	527 (20.2)	796 (20.8)	796 (20.8)	482 (30.4)	482 (30.4)	350 (27.6)	350 (27.6)
Prostatitis	1547 (58.7)	1547 (58.7)	–	–	675 (42.6)	675 (42.6)	–	–
Due to urinary device	19 (0.7)	19 (0.7)	7 (0.2)	7 (0.2)	22 (1.4)	22 (1.4)	9 (0.7)	9 (0.7)
During pregnancy	–	–	93 (2.4)	93 (2.4)	–	–	11 (0.8)	11 (0.8)
Bacteria								
*Staphylococcus aureus*	173 (6.6)	173 (6.6)	73 (1.9)	73 (1.9)	151 (9.5)	151 (9.5)	32 (2.5)	32 (2.5)
*Escherichia coli*	1996 (75.8)	1996 (75.8)	3343 (87.2)	3343 (87.2)	887 (56.0)	887 (56.0)	1006 (79.2)	1006 (79.2)
*Klebsiella pneumoniae*	351 (13.3)	351 (13.3)	349 (9.1)	349 (9.1)	397 (25.0)	397 (25.0)	185 (14.6)	185 (14.6)
*Pseudomonas aeruginosa*	82 (3.1)	82 (3.1)	43 (1.1)	43 (1.1)	102 (6.4)	102 (6.4)	27 (2.1)	27 (2.1)
*Enterococcus*	32 (1.2)	32 (1.2)	26 (0.7)	26 (0.7)	48 (3.0)	48 (3.0)	20 (1.6)	20 (1.6)
Hospital length of stay								
< 7 days	1707 (64.8)	1948 (73.6)	2371 (61.8)	2672 (69.7)	865 (54.6)	1036 (65.4)	664 (51.5)	774 (60.9)
7–30 days	899 (34.1)	679 (25.8)	1426 (37.2)	1145 (29.9)	682 (43.0)	535 (33.7)	596 (46.9)	486 (38.3)
> 30 days	28 (1.1)	17 (0.6)	37 (1.0)	17 (0.4)	38 (2.4)	14 (0.9)	20 (1.6)	10 (0.8)

*Abbreviations*: *UTI* urinary tract infection

^a^ Infection with at least one prior hospitalization (any cause except UTI) in the last 3 months

Patients with HCAI appeared to be slightly older and stayed longer in hospital than CAIs. The distribution of infections were similar to CAIs for men and women, but *Escherichia coli* caused less HCAIs (56.0 and 79.2%, respectively), pointing to the role of other bacteria, notably *Klebsiella pneumoniae* (25.0 and 14.6%), *Staphylococcus aureus* (9.5 and 2.5%) and *Pseudomonas aeruginosa* (6.4 and 2.1%).

For all subgroups, cases stayed longer in hospital than controls, with, for example in men, > 35% of CAI cases vs. < 26% of controls hospitalized 7 days or more, and > 45% vs. 35% for HCAI cases and controls respectively (Table 2).

### Risk factors analyses

#### Community-acquired infections

##### Antibiotic consumption

For men and women, the risk of acquiring an UTI with an ARB increased with the number of previous antibiotic dispensing in the last 3 months, with ORc reaching 4.26 [3.47–5.24] and 3.07 [2.58–3.66] for ≥3 dispensing, respectively (Table 3). No association was found for antibiotic consumption in the 3–12 month exposure window (Additional file 1, Table S4).

**Table 3:** Conditional univariate logistic regression: Risk factors for community-acquired or healthcare-associated urinary tract infection caused by a resistant bacterium compared with a susceptible one, by gender

	Community-acquired infections		Healthcare-associated infections^a^	
	Men (*N* = 5268)	Women (*N* = 7668)	Men (*N* = 3170)	Women (*N* = 2540)
	ORc [CI 95%]	ORc [CI 95%]	ORc [CI 95%]	ORc [CI 95%]
*Antibiotic consumption*				
Number of antibiotic dispensing in the last 3 months				
0	1	1	1	1
1	1.77 [1.53–2.05]	1.59 [1.41–1.78]	1.30 [1.09–1.55]	1.44 [1.18–1.77]
2	2.61 [2.16–3.17]	1.91 [1.63–2.23]	1.83 [1.46–2.28]	1.47 [1.14–1.90]
≥ 3	4.26 [3.47–5.24]	3.07 [2.58–3.66]	2.97 [2.33–3.79]	2.05 [1.59–2.64]
*Urinary tract condition*				
Recurrent UTI^b^				
No	1	1	1	1
Yes	2.04 [1.82–2.29]	1.69 [1.54–1.86]	1.48 [1.27–1.71]	1.45 [1.24–1.70]
Urinary tract disease in the last 12 months				
No	1	1	1	1
Yes	1.08 [0.84–1.39]	1.15 [0.84–1.57]	1.23 [1.05–1.44]	1.21 [0.96–1.54]
Procedure on UT in the last 3 months				
No	1	1	1	1
Yes	1.65 [1.36–1.99]	1.33 [0.95–1.86]	1.09 [0.93–1.27]	1.96 [1.55–2.49]
*Health context*				
Diabetes				
No	1	1	1	1
On insulin	1.14 [0.79–1.65]	0.95 [0.72–1.26]	1.32 [0.91–1.89]	0.70 [0.44–1.10]
Other	0.97 [0.74–1.29]	1.18 [0.93–1.50]	1.02 [0.74–1.41]	0.86 [0.586–1.33]
Immunosuppression				
No	1	1	1	1
Yes	0.98 [0.84–1.14]	1.10 [0.95–1.21]	1.16 [0.98–1.38]	1.05 [0.86–1.27]
Neurological disease				
No	1	1	1	1
Yes	1.00 [0.85–1.17]	1.01 [0.88–1.15]	1.10 [0.92–1.32]	0.98 [0.80–1.19]
Pregnancy				
No		1		1
Yes		1.06 [0.72–1.58]		1.37 [0.72–2.62]
*Antecedent of hospitalization in the last 3 months*				
Length of the longest stay in ICU				
No stay, or stay ≤7 days			1	1
> 7 days			1.67 [1.16–2.38]	1.75 [1.01–3.03]

*Abbreviations*: *ORc* crude Odds ratio, *CI 95* 95% confidence interval, *UTI* urinary tract infection, *UT* urinary tract, *ICU* intensive care unit

^a^ Infection with at least one prior hospitalization (any cause except UTI) in the last 3 months

^b^ at least 3 prior UTIs in the last 12 months

##### Urinary tract conditions

The risk for infection with ARB increased with recurrent UTIs in both men and women. Having undergone a surgical procedure on the urinary tract during the past 3 months was associated with UTI caused by ARB only for men, mainly due to prostate biopsy (ORc 2.55 [1.77–3.68]; data not shown). Surgical procedure nearby the urinary tract was not associated with an increased risk (Additional file 1, Table S4).

##### Associated health conditions

There was no difference between cases and controls regarding pregnancy or comorbidities such as diabetes, immunosuppression, and neurologic diseases.

In multivariate analysis, antibiotic dispensing in the past 3 months was a risk factor for ARB for both men and women, (ORa, 1.61 [1.38–1.88] and 1.47 [1.31–1.66] respectively, for one dispensing) (Table 4). Having undergone at least one procedure on urinary tract in the previous 3 months increased slightly the risk of ARB for men (ORa 1.34 [1.09–1.64]) but not for women. Recurrent UTI remained marginally associated with an increased risk of ARB (ORa 1.21 [1.05–1.40] and 1.23 [1.11–1.38]). These results did not differ according to bacteria or infection site.

**Table 4:** Conditional multivariate logistic regression: Risk factors for community-acquired or healthcare-associated urinary tract infection caused by a resistant bacterium compared with a susceptible one, by gender

	Community-acquired infections		Healthcare-associated infections^a^	
	Men (*N* = 5420)	Women (*N* = 7792)	Men (*N* = 3388)	Women (*N* = 2614)
	ORa [CI 95%]	ORa [CI 95%]	ORa [CI 95%]	ORa [CI 95%]
*Antibiotic consumption*				
Number of antibiotic dispensing in the last 3 months				
0	1	1	1	1
1	1.61 [1.38–1.88]	1.47 [1.31–1.66]	1.30 [1.07–1.57]	1.34 [1.09–1.66]
2	2.28 [1.85–2.81]	1.69 [1.42–2.00]	1.82 [1.43–2.31]	1.34 [1.01–1.76]
≥ 3	3.59 [2.85–4.53]	2.64 [2.18–3.19]	2.94 [2.25–3.83]	1.79 [1.34–2.38]
*Urinary tract condition*				
Recurrent UTI^b^				
No	1	1	1	1
Yes	1.21 [1.05–1.40]	1.23 [1.11–1.38]	1.04 [0.87–1.25]	1.15 [0.95–1.38]
Procedure on UT in the last 3 months				
No	1	1	1	1
Yes	1.34 [1.09–1.64]	1.08 [0.76–.53]	0.86 [0.71–1.04]	1.98 [1.50–2.61]
Urinary tract disease in the last 12 months				
No			1	1
Yes			1.25 [1.04–2.55]	0.78 [0.58–1.03]
*Prior ICU stay in the last 3 months*				
Length of the longest stay in ICU				
No stay, or stay ≤7 days			1	1
> 7 days			1.76 [1.21–2.55]	1.41 [0.80–2.48]

*Abbreviations*: *ORa* adjusted Odds ratio, *CI 95* 95% confidence interval, *UTI* urinary tract infection, *UT* urinary tract, *ICU* intensive care unit

^a^ Infection with at least one prior hospitalization (any cause except UTI) in the last 3 months

^b^ at least 3 prior episodes of urinary tract infection in the last 12 months

#### Healthcare-associated infection

##### Antibiotic consumption

The risk of having an UTI with an ARB increased in both men and women with the number of previous antibiotic dispensing in the last 3 months, with an ORc reaching 2.97 [2.33–3.79] and 2.05 [1.59–2.64] for ≥3 dispensing, respectively (Table 3). Similarly to CAIs, no association was found for antibiotic exposure within the 3–12 month window (Additional file 1, Table S4).

##### Urinary tract conditions

Recurrent UTIs increased the risk of UTI with ARB for both men and women, whereas a prior procedure on the urinary tract during the last 3 months increased the risk of ARB UTI only in women. Urinary tract disease was slightly associated with an increased risk for men.

##### Associated health conditions

No difference between cases and controls was identified for the comorbidities examined, or pregnancy.

##### Previous hospitalization in ICU

An association was found between previous hospitalization in ICU and UTI due to an ARB, when the ICU length of stay exceeded 7 days.

In the multivariate analysis, dispensing of antibiotic in the last 3 months increased the risk of antibiotic resistance, with increasing risk associated with increased number of dispensing, particularly with ≥3 antibiotics delivered (ORa 2.94 [2,25 - 3,84] and 1.79 [1.34–2.38] for men and women respectively) (Table 4). For women, having undergone a surgical procedure on the urinary tract in hospital within the past 3 months increased the risk of ARB (ORa 1.98 [1.50–2.61]). Transition in ICU in the past 3 months was associated with ARB infection for men (ORa 1.76 [1.21–2.55])*.* As for CAIs, these results did not differ with the bacteria or infection site.

#### Specific antibiotic exposure

When focusing on the last antibiotic dispensing (Table 5), the risk of having a CAI due to ARB increased only when a broad-spectrum antibiotic was last dispensed, whether in association with a UTI (ORa 4.23 [3.41–5.26] and 2.15 [1.86–2.49] for men and women respectively), or not (ORa 2.18 [1.91–2.49] and 1.89 [1.69–2.11], respectively). In HCAIs, similar associations were recovered with prior broad-spectrum antibiotics dispensing, although less consistently in women.

**Table 5:** Conditional logistic regression multivariate analysis: adjusted Odds ratio and IC 95% for the last antibiotic dispensing and acquisition of community-acquired or healthcare-associated urinary tract infection caused by a resistant bacteria compared with a susceptible one, by gender, associated UTI and spectrum category

	Community-acquired^a^		Hospital-acquired^b^	
	Men (*N* = 5268) ORa [CI 95%]	Women (*N* = 7668) ORa [CI 95%]	Men (*N* = 3170) ORa [CI 95%]	Women (*N* = 2540) ORa [CI 95%]
No antibiotic dispensing	1	1	1	1
With UTI – Narrow spectrum^c^	2.18 [1.03–1.56]	0.36 [0.04–3.32]	2.34 [0.54–10.12]	3.28 [0.57–19.04]
With UTI – Broad spectrum^c^	4.23 [3.41–5.26]	2.15 [1.86–2.49]	1,91 [1.48–2.47]	1.13 [0.87–1.47]
Without UTI – Narrow spectrum^c^	1.18 [0.87–1.60]	1,30 [1.02–1.67]	0.86 [0.60–1.24]	1.46 [0.99–2.17]
Without UTI – Broad spectrum^c^	2.18 [1.91–2.49]	1.89 [1.69–2.11]	1.87 [1.59–2.20]	1.69 [1.41–2.03]

*Abbreviations*: *ORa* adjusted Odd Ratio, *CI 95* 95% confidence interval, *UTI* urinary tract infection

^a^ Model adjusted on surgical procedures in the last 3 months

^b^ Model adjusted on stays in ICU and surgical hospital procedures in the last 3 months

^c^ Broad and narrow spectrum classification is presented in additional file 1, Table S3

#### Antibiotic ATC3 class exposure

Among the patients prescribed antibiotics, 49%were excluded for interpretation clarity. Excluding these patients, as well as their matched patients, resulted in analysing around 75% of the total sample of patients, exposed and non-exposed to antibiotics (Table 6). When several antibiotic were prescribed, the most frequent couples of antibiotics were broad-spectrum penicillin and cephalosporin or quinolone, or quinolone and cephalosporin.

**Table 6:** Conditional multivariate logistic regression: Risk factors of having a community-acquired or healthcare-associated urinary tract infection caused by a resistant bacterium compared with a susceptible one, by gender and class of antibiotic delivered in the previous 3 months

	Community-acquired^a^		Hospital-acquired^b^	
	Men (*N* = 4028) ORa [CI 95%]	Women (*N* = 5864) ORa [CI 95%]	Men (*N* = 2284) ORa [CI 95%]	Women (*N* = 1884) ORa [CI 95%]
Antibiotics^c^				
No antibiotic	1	1	1	1
Broad spectrum penicillin (BSP)	1.18 [0.96–1.44]	1.34 [1.13–1.58]	1.09 [0.85–1.41]	1.19 [0.90–1.58]
Sulfonamide	3.11 [1.75–5.52]	1.28 [0.80–2.07]	1.37 [0.83–2.27]	0.86 [0.47–1.57]
Cephalosporin	2.53 [1.74–3.68]	1.90 [1.47–2.45]	2.38 [1.53–3.69]	1.60 [1.03–2.47]
Macrolide	0.96 [0.62–1.48]	1.07 [0.76–1.52]	0.66 [0.38–1.15]	1.17 [0.63–2.19]
Quinolone	3.29 [2.46–4.39]	2.52 [1.90–3.34]	2.54 [1.78–3.61]	2.68 [1.60–4.16]
Other antibiotics^d^	2.99 [1.70–5.28]	1.35 [1.04–1.75]	2.65 [1.27–5.52]	0.65 [0.37–1.12]
Cephalosporin and BSP	3.07 [1.64–5.77]	2.66 [1.58–4.47]	3.73 [1.42–9.75]	3.20 [1.23–8.33]
Quinolone and BSP	4.93 [2.94–8.28]	2.11 [1.28–3.46]	6.06 [3.13–11.74]	1.39 [0.75–2.59]
Quinolone and cephalosporin	5.72 [3.08–10.61]	3.38 [1.87–6.09]	2.87 [1.32–6.23]	0.98 [0.38–2.54]

*Abbreviations*: *ORa* adjusted Odd Ratio, *CI 95* 95% confidence interval

^a^ adjusted on surgical procedures in the last 3 months and recurrent UTIs

^b^ adjusted on stays in ICU, surgical hospital procedures in the last 3 months and recurrent UTI

^c^ antibiotic classification is presented in Table S3 of the additional file

^d^ most of “Other antibiotics” were fosfomycin and nitrofurantoins

Overall, three classes of broad-spectrum antibiotics appeared to have an impact on antibiotic resistance: cephalosporins (ORa from 1.60 to 2.53), quinolones (ORa from 2.52 to 3.29) and other antibiotics (ORa from 1.35 to 2.99), which concerned mostly fosfomycin and nitrofurantoins (Table 6). Sulfonamides increased the risk of antibiotic resistance, for men with CAIs (ORa 3.11 [1.75–5.52]. Macrolides were not associated with an increased risk of antibiotic resistance. As can be expected, compared to a single class delivered, the impact of several antibiotics in the previous 3 months was greater, except for women with HCAI. For example, for men with CAI, the ORa associated with receipt of both cephalosporins and quinolones (5.72 [3.08–10.61]) was stronger than specific ORs (2.53 [1.74–3.68] and 3.29 [2.46–4.39] respectively).

## Discussion

This is the first time that risk factors of having UTI with antibiotic-resistant bacteria are examined using the exhaustive French medico-administrative database. Outpatient dispensing of antibiotics within the past 3 months and especially of broad-spectrum antibiotics were consistent risk factors of acquiring UTI caused by an ARB across all categories. The risk declined when the time lag between exposure and infection exceeded 3 months. A urinary tract procedure performed within the last 3 months also increased the risk for women with HCAIs and for men with CAIs. Lastly, long stays in ICU within the last 3 months also increased the risk for men with HCAIs.

Prior exposure to antibiotics has been found to increase the risk of resistance in several studies, both for community and hospital infections [8, 9, 11–16, 25–28]. Specifically, prior administration of quinolones or cephalosporins in the past 3 months was found as a risk factor, in accordance with our findings [9, 10, 15]. Our exploratory analysis also pointed out fosfomycin and nitrofurantoin, prescribed for UTI, as well as antibiotic associations. An estimation of the mean number of dispensing for each class ensured that the impact of a specific class did not reflect a greater dispensing number (Supplementary, Table S5). Inferences from this analysis should however be taken cautiously because almost of a third of patients with antibiotic dispensing were excluded. Excluded patients seemed to be younger than patients included in the analysis (Supplementary, Table S6). Diagnoses distribution was similar in the two populations. There were minor differences in the bacteria species distribution, with no specific trend. The number of dispensing during the previous 3 months seemed to be superior for excluded patients, mostly explained by the fact that most excluded patients had at least two antibiotic classes dispensed. However, this should not impact our results regarding the association between classes and antibiotic resistance. If anything, this would tend to minimize the association between antibiotic dispensing and resistance.

The effect of recurrent UTIs persisted after adjustment for CAI, but not for HCAI. Several prior studies identified previous UTIs as a risk factor [12, 16, 17]. The association weakened after adjustment in multivariate models in some others [10, 13], which could be explained by the obvious correlation between antibiotic use and previous UTI. On the other hand, stays with previous hospitalizations with UTI during the past year were excluded in order to select incident hospitalizations, which resulted in excluding patients having a high rate of ARB infection (rate of 20%, vs 7% in the selected stays), and may have minimized the strength of the association between recurrent UTIs and antibiotic resistance.

As expected, surgical procedures on the urinary tract were identified as risk factors for antibiotic resistance, but only for men with CAI and women with HCAI. This result was consistent with studies where previous urological procedure or presence of a urinary catheter were also identified as risk factor, for hospital and community infections [9, 13, 15, 16]. For women with HCAI, no specific procedure was identified, whereas in men, prostate biopsy mostly explained the association. This suggests the importance of improving infection prevention in these patients, and to follow prophylaxis recommendations.

Prior ICU stays > 7 days in the last months was associated with a higher risk of antibiotic resistance in men. As far as we know, no study has examined the impact of previous ICU stay on UTI due to ARB. However, several studies have shown that staying in an ICU was associated with antibiotic resistance [29–31], and particular emphasis on prevention of infection is warranted in these units.

Neither co-morbidities examined (diabetes, immunosuppression, neurological and urinary tract diseases) nor pregnancy were associated with a higher risk of antibiotic resistance. There is no general agreement on the potential association of ARB with comorbidities [12–14]. A study found renal disease and cancer as risk factors when comparing patients affected with ESBL-p *Escherichia coli* UTI with patients with no UTI [13]. However, when comparing to patients with susceptible *Escherichia coli*, no difference was found regarding renal disease and cancer, and a slightly increased risk with immunosuppressive treatment. Another study reported an association between ARB and immunosuppression in community-acquired UTI, but not with diabetes or pregnancy status [12].

Using a medico-administrative database involves some drawbacks [22]. The two main limitations of the database used are the lack of information on residency in skilled nursing home and on drug administration during hospitalizations. First, because residency in a nursing home was not available, the potential role of this previously identified risk factor [10, 14] could not be examined. Second, because antibiotic delivered during hospitalizations were not recorded in the database, prior antibiotic exposure was identified only through dispensing in the community. However, we tested the hypothesis that each hospitalization with an infection was associated with antibiotic administration, in addition to community exposure. A moderate increase of antibiotic exposures was noted, but no difference was found concerning odds ratios (data not shown). In addition, it is important to note that, in this work, antibiotic consumption was studied using dispensing. Thus, it was assumed that patients actually consumed the antibiotics delivered and that exposure started on the date of dispensing.Third, identification of some co-morbidities required to use algorithms, combining several types of information. However, the risk of misclassification should be similar for cases and controls and most of the algorithms used were constructed by the *Cnam* organism, reviewed by experts of each field, and then improved by *Cnam* organism when necessary. They are freely available to all, which provides standardized definitions for SNDS users. A study estimated the sensitivity of the algorithm for identifying diabetic patients to range from 93 to 99% [32]. Finally, it should be pointed out that CAIs were defined in patients not hospitalized in the past 3 months, which may incur some misclassification of patients. Indeed, a patient with CAI may have nevertheless been exposed to medical environment, ambulatory care surgical procedure, and home care or nursing facilities that were not easily identifiable in the database. Conversely, a patient hospitalized within the past 3 months may still have a community-acquired infection. As expected however, HCAIs affected older patients, having longer stays than those with CAI, and were more frequently caused by nosocomial pathogens, such as *Klebsiella pneumoniae*, *Pseudomonas aeruginosa* or *Staphylococcus aureus*.

Despite its limitations, the large nationwide and comprehensive SNDS database remains a remarkable tool for analysing risk factors. It covers all hospitalizations in France, and all reimbursed drugs dispensed by pharmacy (including antibiotics), as well as biological exams. Hospital diagnosis were validated by Afshar et al., who showed that ICD-10 codes allow to analyze pathogens and resistance markers, and Salhi et al. showed a good coding of infection, with a positive predictive value between 0.98 [0.65–1.00] and 0,93 [0.88–0.98] [33, 34]. Thanks to the access to a wide range of routinely collected detailed information, the SNDS database has been used by other authors to study incidence and prevalence of disease, as well as identify risk factors [20, 35–39]. Our study is the first concerning risk factors of antibiotic resistance in SNDS. The large population included allowed us to stratify the analysis on location of acquisition and sex, and to include a variety of bacteria and resistance codes, unlike most of the studies that focused on ESBL-producing or MDR Enterobacteriaceae UTIs.

## Conclusions

This study confirms the importance of broad-spectrum antibiotic consumption on the risk of UTI with ARB in the last 3 months, and the necessity of controlling antibiotic prescription. It also highlights the importance of prevention during surgical procedure on the urinary tract, and prolonged ICU stays.

## Supplementary Information

**Additional file 1 **Includes all supplementary material. **Supplement 1**. Describes the database and patient selection. **Table S1.** Shows the ICD-10 codes used. **Table S2.** Describes the selected bacteria included and the associated resistance markers. **Supplement S2.** Describes the algorithms for identification of risk factors. **Table S3.** Shows the code lists used for the definition of risk factors. **Table S4.** Shows the conditional univariate logistic regressions of risk factors of having a community-acquired or healthcare-associated urinary tract infection caused by a resistant bacterium compared with a susceptible one, by sex. **Table S5.** Shows the number of antibiotic dispensing during the previous 3 months for each antibiotic class, by type of infection and sex. **Table S6.** Shows the characteristics of patients excluded and included for the analysis of association between antibiotic classes and resistant-bacterial acquisition, according to type of infection and sex.

## Data Availability

The data analysed during the current study are available from the French Caisse National d’Assurance Maladie (CNAM) but legal restrictions apply to the availability these data. Access authorisation is restricted to public interest research and based on the project evaluation. Data are available after obtaining legal authorization from the French national commission governing the data privacy laws (Commission Nationale Informatique et Liberté; CNIL, https://www.cnil.fr/) and from the Health Data Hub (at https://www.health-data-hub.fr).

## Abbreviations

ARB: Antibiotic-resistant bacteria
ATC: Anatomical Therapeutic Chemical classification
CAI: Community infections
CCAM: French classification of clinical procedures (*Classification Commune des Actes Médicaux*)
CIP: French presentation identification code for drugs (*Code Identifiant de Présentation*)
ESBL-p: Extended-spectrum beta-lactamases-producing
HCAI: Health-care associated infection
ICD-10: International Statistical Classification of Diseases and Related Health Problems
ICU: Intensive care units
UTI: Urinary traction infection
PMSI: National hospital discharge database (*Programme de Médicalisation des Systèmes d’Information*)
SNDS: National health data system (*Système National des Données de Santé*)
