# Correction to: Antibiotic prescriptions and risk factors for antimicrobial resistance in patients hospitalized with urinary tract infection: a matched case-control study using the French health insurance database (SNDS)

**DOI:** 10.1186/s12879-021-06329-8

**Published:** 2021-06-29

**Authors:** Marion Opatowski, Christian Brun-Buisson, Mehdi Touat, Jérôme Salomon, Didier Guillemot, Philippe Tuppin, Laurence Watier

**Affiliations:** 1 Epidemiology and Modeling of bacterial Evasion to Antibacterials Unit (EMEA), Institut Pasteur, 25–28, Rue du Dr. Roux, 75724 Paris Cedex 15, France; 2 Center for Research in Epidemiology and Population Health (CESP), INSERM U1018, Paris-Saclay University, UVSQ, Montigny-Le-Bretonneux, France; 3 AP-HP, Paris Saclay, Public Health, Medical Information, Clinical Research, Le Kremlin-Bicêtre, France; 4 French National Health Insurance (Cnam), 50 Avenue du Pr-André-Lemierre, 75986 Paris Cedex 20, France

**Correction to: BMC Infect Dis 21, 571 (2021)**

**https://doi.org/10.1186/s12879-021-06287-1**

Following publication of the original article [1], the authors identified an error in the Supplementary file. The revised and correct version of the Supplementary file is given below:

Furthermore, a typo was found in an author’s affiliation.

The incorrect affiliation name is:

Center for Research in Epidemiology and Population Health ou (CESP), INSERM U1018, Paris-Saclay University, UVSQ, Montigny-Le-Bretonneux, France

The correct affiliation name is:

Center for Research in Epidemiology and Population Health (CESP), INSERM U1018, Paris-Saclay University, UVSQ, Montigny-Le-Bretonneux, France

The original article has been corrected as well.

## Supplementary Information

**Additional file 1: Supplement S1**: Database description and patient selection. **Table S1**: ICD-10 codes used. **Table S2**: Selected bacteria included and associated resistance markers. **Supplement S2**: Algorithms for identification of risk factors. **Table S3**: Code list for definition of potential risk factors. **Table S4**: Conditional univariate logistic regression: Risk factors of having a community-acquired or healthcare-associated urinary tract infection caused by a resistant bacterium compared with a susceptible one, by gender. **Table S5**: Number of antibiotic dispensing during the previous 3 months. **Table S6**. Characteristics of patients excluded and included for analysis of association between antibiotic classes and resistant-bacterial acquisition.
