# Supplementary material for: Antibiotic prescriptions and risk factors for antimicrobial resistance in patients hospitalized with urinary tract infection: a matched case-control study using the French health insurance database (SNDS)
Source: BMC Infect Dis. 2021 Jun 14;21:571. doi: 10.1186/s12879-021-06287-1 (PMC8201676; doi:10.1186/s12879-021-06287-1)
Supplement: Supplementary file 1 — Additional file 1 Includes all supplementary material. Supplement 1. Describes the database and patient selection. Table S1. Shows the ICD-10 codes used. Table S2. Describes the selected bacteria included and the associated resistance markers. Supplement S2. Describes the algorithms for identification of risk factors. Table S3. Shows the code lists used for the definition of risk factors. Table S4. Shows the conditional univariate logistic regressions of risk factors of having a community-acquired or healthcare-associated urinary tract infection caused by a resistant bacterium compared with a susceptible one, by sex. Table S5. Shows the number of antibiotic dispensing during the previous 3 months for each antibiotic class, by type of infection and sex. Table S6. Shows the characteristics of patients excluded and included for the analysis of association between antibiotic classes and resistant-bacterial acquisition, according to type of infection and sex. [file 12879_2021_6287_MOESM1_ESM.pdf]

Additional file 1

Antibiotic prescriptions and risk factors for antimicrobial resistance in patients hospitalized with urinary tract infection: A Matched Case-Control Study using the French health insurance database (SNDS).

Marion Opatowski[a,b], Christian Brun-Buisson[a,b], Mehdi Touat[a,b], Jérôme Salomon[a,b,c], Didier Guillemot[a,b,c], Philippe Tuppin[d], Laurence Watier[a,b]

[a] Epidemiology and Modeling of bacterial Evasion to Antibacterials Unit (EMEA), Institut Pasteur, 25–28, rue du Dr. Roux, 75724 Paris Cedex 15, France

[b] Center for Research in Epidemiology and Population Health (CESP), INSERM U1018, Paris-Saclay University, UVSQ, Montigny-Le-Bretonneux, France.

[c] AP-HP, Paris Saclay, Public Health, Medical Information, Clinical Research, Le Kremlin-Bicêtre, France.

[d] French National Health Insurance (Cnam), 50 avenue du Pr-André-Lemierre, 75986 Paris Cedex 20, France

Corresponding author:

Laurence Watier

Epidemiology and Modeling of bacterial Evasion to Antibacterials Unit

INSERM CESP U1018 / UVSQ / Institut Pasteur

25–28, rue du Dr. Roux, 75724 Paris Cedex 15, France

Email: laurence.watier@inserm.fr

Telephone number: +33 1 45 68 83 01

Table of Contents:

A. Supplement S1: Database description and patient selection
B. Table S1: ICD-10 codes used
C. Table S2: Selected bacteria included and associated resistance markers
D. Supplement S2: Algorithms for identification of risk factors
    1. Antibiotic exposure
    2. Non-hospitalized urinary tract infection
    3. Specific urinary tract disease
    4. Urinary tract surgical procedures
    5. Diabetes
    6. Immunosuppression
        a. HIV or AIDS
        b. Active cancer
    7. Pregnancy
    8. Previous hospitalization in intensive care unit
E. Table S3: Code list for definition of potential risk factors
F. Table S4: Conditional univariate logistic regression: Risk factors of having a community-acquired or healthcare-associated urinary tract infection caused by a resistant bacterium compared with a susceptible one, by gender.
G. Table S5: Number of antibiotic dispensing during the previous 3 months
H. Table S6. Characteristics of patients excluded and included for analysis of association between antibiotic classes and resistant-bacterial acquisition
I. References

A. Supplement S1: Database description and patient selection

The PMSI database covers all hospital stays in French publicly funded and private hospitals. It is the French national hospital discharge database, updated annually and used for reimbursement purposes. Therefore, each hospitalization record is standardized, following the Technical Agency for Hospital Information (ATIH) recommendations. It contains hospital diagnoses and medical procedures performed during each stay. Diagnoses are coded as principal diagnosis (PD: condition requiring hospitalization), related diagnosis (RD: adds information to PD) and secondary associated diagnosis (SAD: complications and co-morbidities potentially affecting the course or cost of hospitalization). This database is linked with all reimbursed outpatient care. A wide range of information is available, such as nature and date of visits, procedures or laboratory tests performed by medical and paramedical professionals, and information on reimbursed drug dispensed (drug name, date and quantity). Moreover, the patient's sociodemographic and medical characteristics (i.e., date of birth, town of residence, health insurance coverage, or specific long-term diseases) are provided. A data quality and consistency plan at several levels ensures homogeneous data processing (ref).

Diagnostics and long-term diseases are coded using the 10th revision of the International Statistical Classification of Diseases and Related Health Problems (ICD-10) [1]. Procedures performed both for inpatients or outpatients are coded with the French classification of clinical procedures (CCAM, *Classification commune des actes médicaux*). Laboratory tests are registered with the French nomenclature of procedures (NABM, *nomenclature des actes de biologie médicale*). Drugs are identified through two classification systems: anatomical therapeutic chemical (ATC) and French presentation identification code (CIP, *Code Identifiant de Présentation*) systems.

To select hospitalizations for urinary tract infection (UTI), hospital admissions of adults (≥18-years-old) occurring between Jan 1st, 2015 and Dec 31, 2017 with a UTI code in PD were selected. Infections were identified, and bacteria thereafter, using codes lists established in collaboration with infectious diseases specialists (Table S1). Incident hospitalizations were then selected, by excluding stays with a previous hospitalization with a UTI code filled in PD, RD or SAD within the past 12 months.

B. Table S1: ICD-10 codes used

Categories	Codes
Principal diagnosis of urinary tract infection	
Acute tubulointerstitial nephritis	N10, N12
Pyelonephritis	N11, N11.1
Renal and perirenal abscess	N13.6, N15.1
Cystitis	N30.0, N30.9
Prostatitis	N41.0, N41.2, N41.3
Due to medical device	T83.5
During pregnancy	O23.0, O23.1, O23.2, O23.4, O86.2
Unspecified	N39.0
Bacteria	
Escherichia coli	B96.2
Klebsiella pneumoniae	B96.1
Staphylococcus aureus	B95.6, A41.0[a], U82.10, U82.100
Pseudomonas aeruginosa	B96.5
Enterococcus	A40.2[a], B95.2
Resistance	
Extended-spectrum β-lactamase producing	U822, U822.0
Methicillin	U82.18, U82.180, U82.10, U82.100
Vancomycin	U83.0, U83.00, U83.1, U83.10
Multiple resistance	U83.71, U83.710, U83.7, U83.78, U83.780
Emerging highly resistant gram-negative bacteria	U83.70, U83.700
Other β-lactams[b]	U82.8, U82.80, U82.9, U82.90
Others[c]	U83.8, U83.80, U83.9, U83.90

[a] When associated with a sepsis code, urinary tract infection bacteria was considered the same as that recorded for sepsis.

[b] not included: penicillin

[c] not included: penicillin and quinolone

C. Table S2: Selected bacteria included and associated resistance markers

	E. coli	*K. pneumoniae*	*S. aureus*	*P. aeruginosa*	Enterococcus
ESBL-producing	✓	✓			
Methicillin			✓		
Vancomycin					✓
Multiple resistance	✓	✓	✓	✓	✓
Emerging highly resistant bacteria					✓
Other β-lactams[a]				✓	
Others[b]				✓	

E. coli = Escherichia coli; K. pneumoniae = Klebsiella pneumoniae; S. aureus = Staphylococcus aureus; *P. aeruginosa* = *Pseudomonas aeruginosa;* ESBL-producing = Extended Spectrum Beta Lactamase producing

[a] not included: penicillins

[b] not included: penicillins and quinolones

D. Supplement S2: Algorithms for identification of risk factors

1. *Antibiotic exposure*

Antibiotic exposures were extracted from the outpatient healthcare claims database, according to the ATC code list (Table S3). In order to exclude antibiotics prescribed for the index UTI, dispensing during the 7 days before hospitalization were not considered. The time interval between antibiotic dispensing and index stay (at least one dispensing in the 8 days - 3 months; > 3 months) and the number of dispensing during the last 12 months (0; 1; 2; ≥ 3) were considered. Because of collinearity between the number of antibiotic dispensing and time interval, a variable combining the two information was created. The last dispensing was given particular attention. Because antibiotic consumption may be associated with an outpatient UTI, and broad spectrum antibiotics may favor the selection of antibiotic-resistant bacteria [2–4], four classes of exposures were considered: associated or not with an UTI, and narrow or broad spectrum antibiotic (spectrum detailed in Table S3). If several antibiotics were delivered simultaneously, the broadest spectrum was considered.

2. *Non-hospitalized urinary tract infection*

An antecedent of non-hospitalized urinary tract infection was defined by:

- a dispensing of an antibiotic specific to urinary tract infection (CIP codes)
- and/or cytobacteriological examination of urine (NABM codes) followed by an antibiotic dispensing (≤ 72h),
- and/or cytobacteriological examination of urine, followed by an antibiotic susceptibility test (NABM codes) (≤ 48h).

Recurrent UTIs was defined as ≥3 UTIs within the last 12 months.

Corresponding codes are shown Table S2.

3. *Specific urinary tract disease*

Prior hospitalization for a *urinary tract or renal disease* in the previous 12 months, such as kidney failure, bladder tumor, or urinary stone were defined from their respective ICD-10 codes (Table S3).

4. *Urinary tract surgical procedures*

Urinary tract surgical procedures were identified using a CCAM code list (Annex, Table S3). The most recent procedure was studied, and classified according to two characteristics: anatomical location (nearby or in contact with urinary tract), and time interval to the index hospitalization (≤ 3, 3-12 months).

5. *Diabetes*

A diabetic patient was defined, according to the French National Health Insurance Agency (*Cnam*) algorithm [5,6] as:

- Having received at least 3 dispensing (on different dates) of oral antidiabetic drugs or insulin during the last 12 months,
- and/or registered as diabetic for long-term condition with ICD-10 codes,
- and/or having been hospitalized with a ICD-code of diabetes (as PD or RD),
- and/or having been hospitalized for a complication of diabetes (as PD or RD) with a diabetes code.

ICD-10 codes of identification:

diabetes codes: E10, E11, E12, E13, E14

complication codes: G59.0, G63.2, G73.0, G99.0, H28.0, H36.0, I79.2, L97, M14.2, M14.6, N08.3

6. *Immunosuppression*

An immunocompromised person was defined as a patient:

- with HIV or AIDS,
- and/or using immunosuppressive treatments,
- and/or with an active cancer,
- and/or using corticoids, with at least 3 dispensing of 20mg/day of prednisolone equivalent during the last 6 months.

Immunosuppressive treatments and corticoids list are available in Table S2. HIV or AIDS and active cancer were identified using French National Health Insurance algorithms, as detailed below.

a. *HIV or AIDS*

According to *Cnam* algorithm [6], a patient was diagnosed with HIV or AIDS when:

- registered for long-term condition with ICD-10 codes of diseases due to HIV/AIDS,

- and/or hospitalized in MCO (medicine, surgery or obstetrics) in PD, RD, or DAS, for HIV/AIDS in the past 2 years,

- and/or hospitalized in MCO during the past 12 months for another diagnosis, with HIV/AIDS as complication or associated morbidity,

- and/or has received at least 3 delivery of at least one drug specific to HIV/AIDS treatment, during the past 12 months,

- and/or has had a medical laboratory procedure specific to the treatment of HIV/AIDS in the past 12 months.

Identification codes:

ICD-10 HIV/AIDS codes: B20, B21, B22, B23, B24, F02.4, Z21

Drug ATC codes: J05AF01, J05AF02, J05AF03, J05AF04, J05AF06, J05AF13, J05AG01, J05AG03, J05AG04, J05AG05, J05AR01, J05AR02, J05AR04, J05AE01, J05AE02, J05AE03, J05AE04, J05AE05, J05AE07, J05AE08, J05AE09, J05AE10, J05AR10, J05AR06, J05AR08, J05AR09, J05AR13, J05AR18, J05AX07, J05AX08, J05AX09, J05AX12.

Medical laboratory procedure codes: 0805, 0806, 4117, 4122

b. Active cancer

According to *Cnam* algorithm [6], a patient is diagnosed with an active cancer when:

- registered for long-term condition with ICD-10 codes of cancer,

- and/or having been hospitalized for cancer during the last 12 months (PD or RD)

For breast cancer, only women are selected, with ICD-10 codes C50 and D05.

For prostate cancer, only men are selected, with ICD-10 codes C61 and D07.5 for hospitalization and/or men aged 40 or more with at least 3 anti-androgenic drug dispensing.

Identification codes:

ICD-10 codes: all ICD-codes beginning with the letter “C” and from D00 to D09

Anti-androgenic d*rugs ATC codes*: L01CD04, L02BX03, L02BX02, L02BB01, L02BB02, L02BB03, L02BB04, L02AE01, L02AE02, L02AE02, L02AE04, G03HA01, L01XX11, L02AA01

7. *Pregnancy*

According to Cnam algorithm [6], a woman was considered as pregnant when aged between 15 and 49 years and hospitalized (in MCO) with a GHM (homogeneous group of patients) code corresponding to delivery or cesarean section within the 9 months following the index hospitalization.

CHM codes : 14C03, 14C06, 14C07, 14C08, 14Z09, 14Z10, 14Z11, 14Z12, 14Z13, 14Z14

8. *Previous hospitalization in intensive care unit (ICU)*

ICU stays > 7 days occurring within the 3 months preceding the index hospitalization were identified for HCAI.

E. Table S3: Code list for definition of potential risk factors

	Codes
Antibiotics[a] (narrow spectrum indicated by *)	
Tetracycline	J01AA02, J01AA04, J01AA05, J01AA08, J01AA12
Amphenicols	J01BA02
Beta-lactams and penicillin	J01CA01, J01CA02, J01CA04, J01CA06, J01CA08, J01CA10, J01CA12, J01CA13, J01CA17, J01CE01*, J01CE02*, J01CE08*, J01CE10*, J01CF02*, J01CF04*, J01CG01, J01CR01, J01CR02, J01CR03, J01CR05
Other beta-lactams	J01DB01, J01DB04, J01DB05, J01DB09, J01DC02, J01DC04, J01DC07, J01DD02, J01DD04, J01DD08, J01DD13, J01DE01, J01DF01*, J01DH02, J01DH03, J01DH51, J01DI01, J01DI02, J01DI54
Sulfonamides and trimethoprim	J01EA01, J01EB02, J01EC02, J01EE01
Macrolides, Lincosamides, Streptogramines	J01FA01*, J01FA02*, J01FA03*, J01FA06*, J01FA07*, J01FA09*, J01FA10*, J01FA11*, J01FA15*, J01FF01*, J01FF02*, J01FG01*
Aminoglycoside	J01GA01, J01GB01, J01GB03, J01GB06
Quinolone	J01MA01, J01MA02, J01MA03, J01MA04, J01MA06, J01MA07, J01MA12, J01MA14, J01MB04*, J01MB07*
Association of antibacterial	J01RA02, J01RA04
Other antibacterial	J01XA01*, J01XA02*, J01XA04*, J01XB01*, J01XC01, J01XD01, J01XD02, J01XD03, J01XE01, J01XX01, J01XX04*, J01XX08*, J01XX09*, J01XX11*
Antecedent of UTI algorithm	
Antibiotics specific to UTI[b]	3400933652395, 3400937374392, 3400939418261, 3400939423524, 3400939463506, 3400939423692, 3400949879311, 3400939423814, 3400939889115, 3400939889054, 3400939889405, 3400939889344, 3400949879250, 3400933209230, 3400949009824, 3400949000524, 3400949006861, 3400949003501, 3400933282387, 3400933459758, 3400933912482, 3400932982530, 3400930057728, 3400932441396, 3400937993050, 3400959069757, 3400931226208, 3400932964499, 3400955368632, 3400930419045, 3400932180011, 3400936267114, 3400956502929, 3400936133419, 3400936266971, 3400936125483, 3400936267343, 3400936267282, 3400937465335, 3400936125544, 3400936797420, 3400936267053, 3400936747104, 3400933085728, 3400955454830, 3400933089221
Cytobacteriological examination of urine[c]	5201
Antibiogram[c]	0269, 0270
Urinary tract disease[d]	A18.1, A54, A54.0-2, A55, A56, A56.0-3, A57-63.8, B90.1, C51-58, C60-60.2, C62-63.9, C64-68.9, D07.1-07.4, D07.6, D28-28.9, D29.0, D29.1, D29.3, D29.7, D30-30.9, D39.7, D39.9, D40, D40.0, D40.7, D40.9, D41-41.9,N04-04.9, N06-06.8, N11, N11.8-11.9, N13-14.4, N15, N15.0, N15.8-16.8, N17-23, N25, N25.8-25.9, N26-30.8, N31-42.9, N45-45.9, N48.1, N49.0-49.1, N50.9, N51.0, N51.8, N70-77.8, N80, N81-82.9, N84, N84.2-84.9, N99, N99.1, N99.5, N99.8-99.9, O23.5, O23.9, O75.3, O85-86.1, O86.3, O04.5, O05.0, O05.5, O06.5, O07.0, O07.5, O90.4, R31-35, R39-39.1
Procedures[e]	
On urinary tract	HJEA001, JACA001-002, JACH001-003, JADA001, JAEA002-003, JAFA001-032, JAFC001-008, JAFC010, JAFC019, JAGA001-006, JAGC001, JAGD001, JAGD002, JAGF001-003, JAHA001, JAHB001, JAHC001, JAHH001-002, JAHJ002, JAHJ004-007, JAKD001, JAMA001-002, JAMB001, JANE002, JANE005, JANH001-002, JANJ002, JANM001-002, JAPA001, JAPC018, JAQH003, JASA001, JBCA001, JBFA001-002, JBFC001, JBFE001, JBHE001, JBMA001, JBMC001, JBNE004, JBPE001-002, JBPG001, JBPH001, JBQE001, JBQG001-002, JBQH001-003, JCAD001, JCAE001, JCAF001, JCAH001, JCCA002-003, JCCA005-014, JCCB001, JCCC001-003, JCCC003, JCEA001-0006, JCEC011, JCEE001, JCFA001-010, JCFC001, JCFD001, JCFE001-003, JCFF001, JCGA001-003, JCGC001-003, JCGE001-006, JCGG001, JCGH001-005, JCKA001-002, JCKD001, JCKE001-002, JCKH001, JCLD001, JCLE001-004, JCLH001, JCMA001-005, JCND001, JCNE001, JCNE002-004, JCNF001, JCNM001-002, JCPA001-003, JCPC001-002, JCPD001, JCPE001-002,

	JCQE001-004, JCSA001-005, JDAD001, JDCA001-004, JDCC016, JDCJ001, JDDA002-008, JDDB005, JDDB007, JDDC002, JDFA001-025, JDFC001, JDFC023, JDFE001-003, JDGE001, JDHE001-003, JDJD001-002, JDJE001, JDKD001-002, JDLA001, JDLD001-002, JDLE332, JDLF001, JDMA001-003, JDNE001, JDNM001, JDPA001-002, JDPE001-002, JDQD001-006, JDQD008-009, JDQE001-003, JDQF001-002, JDQH001-003, JDSA001-011, JDSC024, JEAA001, JEAD001, JEAH001, JECA001-002, JECE001, JEEE001, JEFA001-012, JEFE001, JEFE003-005, JEGA002-005, JEGB005, JEGE001-004, JEHD001, JEHE001-002, JEKA002, JEKA005-010, JEKB006, JEKE001, JELA001-004, JELB003, JELD001, JELE001-003, JEMA001-007, JEMA009-023, JENE001, JEPA001, JEPA006-007, JEPD001-002, JEPE001-002, JEPH001, JEQD001, JEQD002, JESA001, JESA003-005, JFFA001-005, JFFA008-09, JFFA011, JFFA013, JFFA016, JFFA018, JFFA019, JFFA022, JGFA005-007, JGFA009, JGFA011, JGFA014, JGFA016, JGFC001, JGFE004, JGFE023, JGFE365, JGHB001-002, JGHD001, JGHJ001-002, JGJB001-003, JGNE171, JGNJ900, JHQB001, JHQP002, JMBA001, JMDA001, JRGA001-004, JRGC001, JRPA001, JRQD001, JRQP001, JRRD001, JZMA002-002, JZQD001
Proximity to urinary tract	HJMA001, HKEA001, HKMA006, HKPA006, HPQC001, JFFA012, JFFC001, JGFA008, JGFC045, JGQH001, JGQH003, JGQH004, JHAA001-005, JHBA001, JHCA001-002, JHCA004-006, JHDA001, JHEA001-012, JHEP001-002, JHFA001-019, JHFC001, JHGA00-003, JHJA001, JHJB001-003, JHKA002-004, JHLA001-005, JHLB001-002, JHMA001-008, JHMB001, JHNP001, JHPA001-003, JHQH001, JHSA001-002, JHSB001, JJCC002, JJLE001-002, JJQC001, JKBA001, JKFA002, JKFA005-008, JKFA011, JKFA018, JKFA020-021, JKFA023, JKFA025-026, JKFA030-031, JKFA033, JKFD001-002, JKFE001-003, JKGD002, JKGE002, JKND001-004, JKNE001, JKPC001, JKPD002, JKPE001-002, JKPJ001-002, JKQE001-002, JLAD001, JLCA003, JLCA005-009, JLDA002, JLFA001-002, JLFA004, JLFD001-002, JLGD001, JLGE001, JLJA002, JLJB001, JLJD001, JLLD001, JLMA001-005, JLMC001, JLMD001, JLND001-002, JLPA001, JLQE001-002, JLSD001, JMCA001-006, JMEA001-002, JMFA001-010, JMMA001-002, JMMA004-005, JMPA001-005, JMPP001, JMQP001, JZMA001
Immunosuppression algorithm	
immunosuppressive treatment[a]	L04, L01A, L01B, L01C, L01D, L01X, L03AB, J05AE01, J05AE02, J05AE03, J05AE04, J05AE05, J05AE06, J05AE07, J05AE08, J05AE09, J05AE10, J05AF01, J05AF02, J05AF03, J05AF04, J05AF06, J05AF13, J05AG01, J05AG03, J05AG04, J05AG05, J05AR01, J05AR02, J05AR04, J05AR06, J05AR08, J05AR09, J05AR13, J05AR18, J05AX07, J05AX08, J05AX09, J05AX12,
Corticosteroids[a]	H02AB01, H02AB02, H02AB04, H02AB06, H02AB07, H02AB08, H02AB09, H02AB10

UTI = Urinary tract infection

[a] ATC code: Anatomical Therapeutic Chemical code

[b] CIP code: Presentation identification code, unique national drug specialty registration code

[c] NABM code: French nomenclature of clinical pathology procedures code

[d] ICD-10 code: International Classification of Diseases 10th Revision

[e] CCAM code: French medical classification of clinical procedures

F. Table S4: Conditional univariate logistic regression: Risk factors of having a community-acquired or healthcare-associated urinary tract infection caused by a resistant bacterium compared with a susceptible one, by gender.

	Community-acquired infections		Healthcare-associated infections	
	Men (N=5268)	Women (N=7668)	Men (N=3170)	Women (N=2540)
	ORc [CI 95%]	ORc [CI 95%]	ORc [CI 95%]	ORc [CI 95%]
Antibiotic consumptiona[a]				
Number of antibiotic dispensing				
0	1	1	1	1
1	1.14 [0.97 – 1.33]	1.13 [0.99 – 1.29]	1.09 [0.88 – 1.34]	1.17 [0.93 – 1.48]
2	1.42 [1.19 – 1.70]	1.44 [1.24 – 1.67]	1.12 [0.91 – 1.39]	1.32 [1.03 – 1.70]
≥ 3	2.48 [2.15 – 2.87]	2.04 [1.81 – 2.29]	1.87 [1.55 – 2.26]	1.66 [1.36 – 2.03]
Delay between dispensing and stay				
no dispensing, or ≤ 7 days	1	1	1	1
8 – 90 days	2.43 [2.12 – 2.79]	2.03 [1.82 – 2.30]	1.70 [1.43 – 2.02]	1.68 [1.39 – 2.04]
> 90 days	1.00 [0.85 – 1.16]	1.13 [1.00 – 1.28]	0.96 [0.79 – 1.16]	1.11 [0.90 – 1.37]
Urinary tract condition				
Surgical procedure [a]				
No	1	1	1	1
On urinary tract	1.46 [1.26 – 1.70]	1.36 [1.13 – 1.65]	1.16 [1.00 – 1.34]	1.63 [1.31 – 2.03]
Nearby urinary tract	1.20 [0.46 – 3.13]	0.87 [0.60 – 1.26]	0.72 [0.23 – 2.27]	0.78 [0.44 – 1.39]
Delay between procedures and stay				
No procedure	1	1	1	1
≤ 3 months	1.70 [1.40 - 2.05]	1.27 [0.94 - 1.73]	1.12 [0.96 – 1.31]	1.75 [1.39 - 2.20]
> 3 months	1.22 [1.00 – 1.50]	1.24 [1.02 – 1.51]	1.28 [0.96 - 1.69]	0.96 [0.66 - 1.37]
Prior ICU stay in the last 3 months				
No stay			1	1
1 or 2 days			1.08 [0.80 - 1.45]	1.28 [0.88 - 1.89]
3 - 7 days			1.22 [0.94 - 1.59]	1.16 |0.82 - 1.62]
> 7 days			1.71 [1.20 - 2.45]	1.79 [1.03 - 3.10]

ORc = Crude OR; CI 95%, 95% confidence interval, ICU = intensive care unit

[a] In the past 12 months

G. Table S5. Number of antibiotic dispensing during the previous 3 months

	Community-acquired				Hospital-acquired			
	Men		Women		Men		Women	
	Mean	SD	Mean	SD	Mean	SD	Mean	SD
Antibiotic class								
Broad spectrum penicillin (BSP)	1.18	0.51	1.15	0.44	1.24	0.59	1.22	0.52
Sulfonamide	1.45	0.71	1.51	0.75	1.47	0.76	1.40	0.74
Cephalosporin	1.24	0.51	1.17	0.46	1.17	0.42	1.28	0.60
Macrolide	1.23	0.51	1.16	0.41	1.17	0.31	1.25	0.53
Quinolone	1.26	0.53	1.19	0.51	1.27	0.56	1.13	0.37
Other antibiotics	1.74	1.21	1.42	0.79	1.20	0.47	1.42	0.78
Cephalosporin and BSP	2.58	0.63	2.41	0.72	2.43	0.73	2.65	1.29
Quinolone and BSP	2.48	0.86	2.61	0.89	2.56	0.87	2.52	0.89
Quinolone and cephalosporin	2.64	0.71	2.48	0.83	2.63	1.08	2.50	0.86

When two classes of antibiotics were dispensed in the 3 months, each antibiotic class counted for one dispensing, even if the two antibiotics were delivered at the same time. The minimum number in this case is 2.

H. Table S6. Characteristics of patients excluded and included for analysis of association between antibiotic classes and resistant-bacterial acquisition

	Community-acquired								Hospital-acquired							
	Men				Women				Men				Women			
	Excluded (N=1240)		Included (N=4028)		Excluded (N=1804)		Included (N=5864)		Excluded (N=886)		Included (N=2284)		Excluded (N=656)		Included (N=1884)	
	N	%	N	%	N	%	N	%	N	%	N	%	N	%	N	%
Age (years)																
Mean (SD)	69.6	15.2	71.4	15.2	67.0	20.7	67.9	22.4	72.8	13.1	75.8	12.4	71.9	17.4	75.2	17.3
Median (IQ)	72.0	21.0	73.0	20.0	72.0	30.0	75.0	33.0	74.0	18.0	78.0	17.0	75	22	80.0	21.0
Diagnostic																
Tubulo-interstitial nephritis	150	12.10	586	14.55	1018	56.43	3684	63.27	142	16.03	390	17.08	318	48.48	1036	54.99
Pyelonephritis	6	0.48	40	0.99	32	1.77	152	2.60	6	0.68	14	0.61	24	3.66	42	2.23
Renal and perirenal abscess	22	1.77	88	2.18	56	3.10	216	3.63	26	2.93	52	2.28	22	3.35	56	2.97
Cystitis	58	4.68	132	3.28	214	11.86	504	8.19	50	5.64	132	5.78	90	13.72	212	11.25
Unspecified UTI	280	22.58	774	19.22	438	24.28	1154	19.70	284	32.05	680	29.77	192	29.27	508	26.96
Prostatitis	714	57.58	2380	59.09	0	0.00	0	0.00	368	41.53	982	43.99	0	0.00	0	0.00
Due to urinary device	10	0.81	28	0.70	4	0.22	10	0.17	10	1.13	34	1.49	8	1.22	10	0.53
During pregnancy	0	0.00	0	0.00	42	2.34	136	1.92	0	0.00	0	0.00	2	0.30	20	1.17
Bacteria																
Staphylococcus aureus	46	3.71	300	7.45	36	2.00	110	1.88	66	7.45	236	10.33	10	1.52	54	2.87
Escherichia coli	959	76.61	3042	75.52	1422	78.82	5264	89.78	494	55.76	1280	56.04	494	75.30	1518	80.57
Klebsiella pneumoniae	188	15.16	514	12.76	292	16.19	406	6.92	222	25.06	572	25.04	124	18.90	246	13.06
Pseudomonas aeruginosa	44	3.55	120	2.98	32	1.77	54	0.92	76	8.58	128	5.60	12	2.83	42	2.23
Enterococcus	12	0.97	52	1.29	22	1.22	30	0.51	28	3.16	68	2.98	16	2.44	24	1.27
Hospitalization lenght of stay																
< 7 days	888	71.61	2757	68.45	1193	66.13	3850	66.65	573	64.67	1328	58.14	392	59.76	1036	54.99
7 - 30 days	343	27.66	2135	30.66	600	33.26	1971	33.61	303	34.20	914	40.02	256	39.02	826	43.84
> 30 days	9	0.73	36	0.89	11	0.61	43	0.73	10	1.13	42	1.84	8	1.22	22	1.17
Number of dispensing in the previous 3 months[1]																
Mean (SD)	2.55	1.40	1.43	0.75	2.50	1.39	1.35	0.68	2.39	1.29	1.39	0.74	2.48	1.27	1.40	0.74
Median (IQ)	2.00	2.00	1.00	1.00	2.00	2.00	1.00	1.00	2.00	2.00	1.00	1.00	2.00	1.00	1.00	1.00

Excluded patients were patients with several classes of antibiotic delivered during this period (except the three most frequent combinations) or antibiotics rarely dispensed (<1% of studied sample), and their matched patients.

When two classes of antibiotics were dispensed in the 3 months, each antibiotic class counted for one dispensing, even if the two antibiotics were delivered at the same time. The minimum number in this case is 2.

[1] among patients with antibiotic prescription in the previous 3 months

I. References

[1] WHO. International Statistical Classification of Diseases and Related Health Problems, 10th Revision (ICD-10). 2nd ed. 2015. http://apps.who.int/classifications/icd10/browse/2015/en.

[2] Yilmaz E, Akalin H, Özbey S, Kordan Y, Sinirtaş M, Gürcüoglu E, et al. Risk Factors in Community-Acquired/Onset Urinary Tract Infections Due to Extended-Spectrum Beta-Lactamase-Producing Escherichia coli and Klebsiella pneumoniae . J Chemother 2008;20:581–5. https://doi.org/10.1179/joc.2008.20.5.581.

[3] Ena J, Arjona F, Martínez-Peinado C, del mar López-Perezagua M, Amador C. Epidemiology of urinary tract infections caused by extended-spectrum beta-lactamase-producing Escherichia coli. Urology 2006;68:1169–74. https://doi.org/10.1016/j.urology.2006.08.1075.

[4] Boix-Palop L, Xercavins M, Badía C, Obradors M, Riera M, Freixas N, et al. Emerging extended-spectrum β-lactamase-producing Klebsiella pneumoniae causing community-onset urinary tract infections: a case–control–control study. Int J Antimicrob Agents 2017;50:197–202. https://doi.org/10.1016/j.ijantimicag.2017.03.009.

[5] Fuentes S, Cosson E, Mandereau-Bruno L, Fagot-Campagna A, Bernillon P, Goldberg M, et al. Identifying diabetes cases in health administrative databases: a validation study based on a large French cohort. Int J Public Health 2019;64:441–50. https://doi.org/10.1007/s00038-018-1186-3.

[6] Constantinou P, Tuppin P, Fagot-Campagna A, Gastaldi-Ménager C, Schellevis FG, Pelletier-Fleury N. Two morbidity indices developed in a nationwide population permitted performant outcome-specific severity adjustment. J Clin Epidemiol 2018;103:60–70. https://doi.org/10.1016/j.jclinepi.2018.07.003.
